# Human rights education in patient care

**DOI:** 10.1186/s40985-017-0061-8

**Published:** 2017-07-11

**Authors:** Joanna N. Erdman

**Affiliations:** MacBain Chair in Health Law and Policy, Schulich School of Law, Dalhousie University, Halifax, Nova Scotia B3K6S2 Canada

**Keywords:** Human rights, Education, Health professions, Patient care, Advocacy, Systemic reform

## Abstract

This article explores how human rights education in the health professions can build knowledge, change culture, and empower advocacy. Through a study of educational initiatives in the field, the article analyzes different methods by which health professionals come to see the relevance of human rights norms for their work, to habituate these norms in everyday practice, and to espouse these norms in advocacy for social justice. The article seeks to show the transformative potential of education for human rights in patient care.

## Background

Human rights education seeks to embed human rights norms into core social institutions. From the recognition that health care systems are core social institutions, human rights were introduced into health professional higher education, that is, the formal curriculum in schools of medicine, nursing and public health. Health professional education, however, presents challenges for the theory and practice of human rights education. There are administrative challenges, such as the difficulty of introducing non-clinical subjects into professional curricula, but there are also substantive challenges related to the very concept of human rights in patient care. This concept, human rights in patient care, refers to the application of legally enforceable human rights in the context of patient care, defined as the provision of services by health providers for the benefit of patients inside any health care setting, including hospitals, clinics, outreach facilities, places of detention or private homes [1]. The challenges of protecting and promoting human rights in patient care include the fact that patient care settings are often closed and intimate spaces, where providers and patients engage in a deeply personal and vulnerable way, all of which complicate the dynamics of identifying and remedying human rights violations especially in the context of structural inequalities, such as power imbalances between providers and patients, particularly those from marginalized social groups [2]. Moreover, many human rights violations in patient care stem from professional norms and institutional structures, such as defenses of certain practices on grounds of administrative efficiency, behavioral modification, or medical necessity, which present unique challenges for systemic reform as human rights law must compete with other powerful normative systems that regulate health professional conduct and contexts [2].

This article sets out to explore how education can lead health professionals to see the relevance of human rights norms for patient care, to habituate these norms in their everyday practices of patient care, and to espouse these norms in social justice advocacy to improve the lives and health of their patients. The concept of human rights in patient care sets an ambitious frame for thinking about the transformative potential of human rights education in the health professions because it is so expansive in its scope. The concept includes the interpersonal relationship between patient and provider in the delivery of healthcare, but also reaches the systemic factors and state responsibility that shape the health care encounter itself [1].

## Human rights education in the health professions: post-apartheid South Africa

Education systems usually perpetuate themselves by reference to tradition, but in times of political transition and often in a deliberate effort to break with the past, there is a greater openness to new influences. Systemic human rights abuse at the hands and with the acquiescence of health professionals characterized the regime of apartheid South Africa [3]. In 1997, the South African Truth and Reconciliation Commission found that:

the health sector through apathy, acceptance of status quo and acts of omission, allowed the creation of an environment in which the health of millions of South Africans was neglected, even at times actively compromised … [in violation] of human rights [4].

International attention has been directed to the specific prohibition against health professionals’ use of expert knowledge and skill in acts of torture and other forms of cruel, inhuman, and degrading treatment in prisons, police, and armed forces [5]. The Commission in South Africa, however, drew particular attention to the routine human rights violations of informed consent, confidentiality, and non-discrimination in the everyday practices of health professionals simply ‘doing their jobs’ in a deeply flawed system. Acknowledging that professional culture develops in the earliest of training, the commission identified education as a key factor that corrupted the health sector, and thus a key measure for its reform. The commission called for the integration of human rights into all health professional education.

In the 1970s, the human rights movement broadened from a strictly legal into a social enterprise with education at its core [6]. In an effort to target systemic violations, human rights education was promoted as a means to embed human rights within social institutions such as health care systems. By the 1990s, human rights education matured into a distinct field of theory and practice [7–11]. It also began to attract targeted political endorsement as reflected in the U.N. Decade of *Human Rights Education* (1995–2006) and its Programme of Action [12, 13]. At the conclusion of the decade, the *Office of the High Commission for Human Rights* established the *World Programme for Human Rights Education* to promote a common understanding of the basic principles and methodologies of human rights education, with resolutions and plans of action for its effective implementation [14]. In 2011, the U.N. General Assembly adopted the *Declaration on Human Rights Education and Training* in an effort to *set* international standards for the field [15]. Emboldened by these initiatives, governments, education sectors, and civil society generated training materials, teaching tools, and other resources on human rights education.

During this time, human rights education in higher education also advanced [16]. From 1968 to 2000, more than 140 universities in 59 countries established academic chairs, research centers, and programs for human rights [17]. In 2006, the Harvard School of Public Health and the University of New South Wales collected teaching materials from more than 30 health and human rights courses offered in academic faculties worldwide [18]. This collection is now housed at the University of Southern California, Institute for Global Health [19].

The integration of human rights into health professional education has had a more measured progress, despite strong international endorsement of human rights as a core component of professional competence [20, 21]. In 2005, the U.N. Special Rapporteur on the Right to Health described human rights education as “an essential starting point for equipping health professionals with the knowledge and tools to empower them to promote and protect human rights” (para 11) [22]. The Special Rapporteur further recognized the many excellent human rights training manuals, courses, and other initiatives for health professionals developed in recent years. Among the most documented is the *Health and Human Rights Programme* at the *School of Public Health and Family Medicine* in Cape Town, South Africa, developed in response to the Truth and Reconciliation Commission’s call for educational reform in the country [23–29]. The field, however, continues to grow [30–34]. There is now a robust English language literature on human rights initiatives in health professional education worldwide, with a commitment to theorizing and evaluating their effectiveness. This literature grapples with questions of why and how to teach human rights in the health professions, exploring objectives and methods of practice. Felisa Tibbitts, a leading scholar of human rights education, identifies these questions as critical to the field as a whole. To move beyond a collection of interesting and discrete programs, she explains, there needs to be a theory about human rights education: its objective and methodology—where to invest energies and funding and where to create new opportunities [35].

Human rights education is not a rigorously defined term, reflecting and perhaps encouraging the many different ways in which it is conceived and practiced [36]. Nonetheless, there is broad consensus on three basic objectives of human rights education: to educate *about* human rights, to educate *through* human rights, and to educate *for* human rights. These objectives, albeit differently articulated, are a common analytical framework in the field [15, 37]. The first objective seeks to impart knowledge *about* human rights, namely, the laws and institutions designed for their protection. Building knowledge is a traditional objective of higher education. The second and third objectives of human rights education are less orthodox. To educate *through* human rights is to change the values, beliefs, and behaviors of an institution, that is, to enculturate human rights or to build a rights-respecting culture. To educate *for* human rights is to empower advocacy for social justice, to build the skills and capacity to act with and on behalf of those whose rights are denied or neglected.

Structured by these objectives, this article explores how human rights education in the health professions can build knowledge, change culture, and empower advocacy. Through an analysis of teaching materials and methods used in each objective, the goal of the article is to generate a theory of human rights education in the health professions and to thereby guide and encourage continued growth of the field. The analysis is based on an interpretive review of the academic English language literature on human rights curricular initiatives in health professional education (by the constraints of the literature mainly medicine, nursing, and public health) in formal diploma or university degree programs, excluding post-graduate training. Articles reviewed were limited to those that describe in sufficient detail the objectives and methods of an initiative. [24, 25, 27, 28, 38–61]. Initiatives were then selected for analysis that illustrated key insights and cross-cutting themes as informed by the general literatures on human rights and health professional education theory and practice.

In interpretive reviews, it is neither possible nor desirable to specify a precise review question, explicit search categories, and tightly specified inclusion criteria such that the search methods can be reproduced [62]. Human rights education is not a rigorously defined term, but rather reflects and encourages many different forms of practice. The review was designed to capture this diversity and to allow concepts and a theory of human rights education to emerge from it. The review was therefore highly inductive and iterative, crucially involving judgment and interpretation. It combined a number of strategies including searching electronic databases, but also reference chaining and using the author’s legal expertise to identify and draw insights from relevant materials in adjacent fields.

These methods are appropriate to the emergent and exploratory aims of this interpretive review, which seeks to generate theory from a large and complex literature and to thereby provide a more formalized, insightful way to think about human rights education in the health professions. The review does not seek to provide a history of the field, nor a comprehensive assessment of human rights initiatives in health professional education on a global scale.

The review is also limited to health professional education rather than a broader category of health provider or worker trainings because of the unique relationship between health professions and the state [63, 64]. First, through legal regulation of licensure and practice, including formal education, the professions are a key site of state power and therefore a key object of human rights law, which imposes obligation on the state in its exercise of power. Second, professions are characterized by their commitment to serve the collective needs of society or the public interest, a commitment integral to the privileges they enjoy from the state, but also integral to the role they can play in the protection and promotion of human rights within the state. The health professions, in other words, are unique mediators between patients and the state, the relationship at the center of human rights law.

## Human rights education as building knowledge

The general practice of human rights education remains firmly rooted in law. The texts of international and regional human rights treaties, and their interpretation by courts of law and other legal institutions, continue to serve as the fundamental content of human rights education, including in the health professions. To make this legal content more accessible for diverse audiences, however, there are simplified and organized collections of health and human rights law intended for use in curricular initiatives. The *Health and Human Rights Resource Guide* and the *Global Health and Human Rights Database*, for example, pair health-related provisions of international treaties with key interpretations and plain language case summaries [65, 66].

These resources make international human rights law more accessible, but not necessarily more relevant for health professional education. They do not answer the questions: why should health professionals learn about international law? How does it affect their delivery of patient care? Relevance is a concern in human rights education, the need to firmly ground human rights in real problems that local actors, including health providers, face on a daily basis [30, 32, 67]. The most immediate relevance of law for health practice is liability, the threat of legal sanction driving behavior change. The UN Special Rapporteur on the Right to Health once noted that most health professionals whom he met had not heard of the right to health, and if they had, they worried it was something that would get them into trouble [68]. International human rights law, however, does not directly sanction health professionals except where they act as agents of the state. International law applies indirectly to patient care through state responsibility for the regulation of health professions and their practice.

In answer to this question of relevance, many human rights initiatives seek to *domesticate* international law by showing how its norms are reflected in and therefore enforceable through national law, including constitutional rights, statutory regulation and even ‘soft law’ such as patient charters and professional codes. Domestic translation, for example, is a key feature of the *Open Society Foundations* (OSF) initiative to build and strengthen capacity for human rights education on patient care in Eastern Europe and Central Asia [47]. In this initiative, OSF commissioned systematic reviews of national laws and regulations related to patient care, which were then cross-referenced against international human rights standards [69]. Although these reviews were intended for lawyers in the region, they proved a valuable resource and are now used for teaching in health professional programs [47].

Teaching international human rights law through local regulatory systems imparts an important lesson about the nature of human rights, namely, that despite universal legal frameworks, human rights are read into and become relevant in local contexts in remarkably different ways [70]. Human rights initiatives in health professional education may thus endeavor to *contextualize* the human rights content, even sometimes, presenting the dynamic between international law and local culture as something itself worthy of study. Strict education transfer or borrowing, the use of generic curricular legal content, is seldom seen in practice even when initiatives are supported by or developed with international actors [71]. In Cambodia, for example, a human rights initiative in health professional education explored the relationship between Buddhist values and human rights [53]. In national contexts with communist or socialist histories, social and economic rights tend to resonate more with the vestiges of these political traditions in national law, such as constitutional rights to health care as a state entitlement [72]. The lesson is that the norms of international human rights law can be accommodated into different legal and political traditions, and it is this resonance that often makes human rights relevant and meaningful in a local context [73]. At the same time, the contrast between international and national law can sometimes offer the more powerful insight. In Ukraine, for example, social and economic rights provide a discourse to support state entitlements to health care against a collapsing public system, while civil and political rights sustain critiques against state abuse of psychiatric institutionalization in the Soviet era [74].

Simple knowledge of law, however, whether international or national, has its limitations in health professional education. Legal rules can teach students what human rights require in patient care, but they rarely generate more complex understandings of why. In a UK study on the integration of law in medical education, for example, students expressed a preference for law over ethics because they saw law as offering more clear-cut answers [75]. Such an impression of law often comes from the method by which it is taught. Outsider disciplines like law may seek legitimacy in health professional education by embracing its positivist methods, for example, teaching human rights as though they were akin to the abstract, generalized knowledge of the basic sciences [76]. The provisions of international human rights treaties, however, are written in open and broad language, and mere knowledge of them rarely if ever provides clear-cut answers to the real conflicts health providers face in practice. Human rights, like all law, require interpretation.

The study of case law, for this reason, becomes important in human rights education. The jurisprudence of the European Court of Human Rights, for example, offers a rich source of educational content. Its case law covers a range of patient care issues such as mistreatment in reproductive and mental health, access to medicines, information disclosure, informed consent, and physician-assisted death [77–80]. Learning law through the study of its application, what is known as the case method in legal education, offers a number of advantages. Not only does it contextualize the rules of law within a set of facts but it also requires students to engage with legal reasoning, that is, the analytic work of moving between fact and law [81]. Students are asked to follow the logic of legal argument, to identify and perhaps contest the facts and assumptions that guide it, and to assess the ultimate judgment reached. These are critical analytical skills that students then carry with them, a new capacity to evaluate the human rights implications of an institutional policy or a clinical practice. Moreover, by reasoning together but arriving at different conclusions about the correctness of a legal judgment, students reveal the possibility of multiple rather than singular answers in law. This again communicates something about the nature of law, namely, that the content of international human rights law is dynamic, evolving over time through ongoing and collective interpretation. To understand the nature of law in this way opens up a new relationship between the health professions and law. Rather than being merely subject to law, they have a role to play in the making of law. After all, it is ultimately health providers who will give true meaning to the abstract standards of human rights law through their interpretation in the daily practice of patient care.

The case method yet has its shortcomings. Most importantly for health professional education, legal cases present a fixed and often simplistic account of the circumstances that gave rise to human rights allegation or violations and provide only one account of their resolution [82]. Legal cases can rarely capture the complexity of unfolding conflicts, competing interests, and institutional constraints that underlie human rights violations. Against the retrospective study of legal cases, problem solving through human rights case studies is thus a valued method in health professional education. Constructed from a diverse set of materials—including law, professional guidance, clinical notes, social science studies, and first person testimonies—case studies are designed to reflect a range of perspectives on a human rights controversy and to generate multiple pathways to its resolution. In the Cambodian initiative, for example, a case study focused on the role of health professionals in torture included excerpts from a Khmer Rouge interrogator’s manual in an effort to expose the mindset of those who torture and the political ideology behind state torture [53]. The objective of case studies is to present international human rights law not only as a standard against which to assess action but also as a framework within which to understand, to work through, and ultimately to resolve the conflicts that breed violations.

Case studies in dual loyalty, where health professionals act for persons and purposes other than the wellbeing of their patients, present immediate and relevant conflicts for engaging in this work [1]. A curriculum module in a new medical school in northern Israel, for example, asked students to reflect on contemporary ethical issues against the abuses of medicine during the Third Reich, an effort to better see and understand the professional power of medicine and the capacity for its abuse [58]. Apart from the egregious cases of medical abuse for repressive state ends, dual loyalty conflicts can underlie more routine human violations in patient care. The global economic deregulation of health care, for example, where commercial forces drive delivery practices and private profits measure quality of care can breed systemic conflicts of interest in patient care [83]. Dual loyalty conflicts, however, do not always represent violations of human rights in themselves. Rather international human rights law, in many cases, justifies restrictions on patient rights for any number of state interests, including public health and criminal justice. This is why dual loyalty conflicts present good case studies. They reveal human rights violations as not the arbitrary actions of wayward individuals, but rather, as the predictable outcomes of the conflicting interests and incentives in health care systems [84].

The Special Rapporteur on the Right to Health has explicitly acknowledged these complex pressures that underlie human rights violation in patient care, and that often leave health providers believing or feeling they have no choice but to violate human rights [22]. Providers are constrained in their ability to provide quality care where they cannot work under decent conditions with professional independence [1]. The failure to acknowledge these institutional and structural barriers frustrates the effectiveness of human rights education [25, 85]. Holding healthcare providers responsible for circumstances they cannot change, moreover, may engender backlash against human rights as unfairly punitive [86]. Under international law, governments are required not only to respect human rights in patient care but also to fulfill the conditions necessary for their realization [87]. This is where international law becomes directly relevant to health professionals because, as explicitly stated by the Special Rapporteur, the right to health imposes obligations of fulfillment on the state to “build[] an environment that supports the adoption of rights-based approaches by the health professional community” (para 16) [22]. Safe working conditions and adequate health resources are human rights entitlements that health professionals can rightly claim from the state. This is the most critical knowledge gained from a human rights analysis in patient care: changing health care practice requires changing the health care institution that sustains it.

## Human rights education as changing culture

A second objective of human rights education is to enculturate human rights within an institutional context, that is, to have health professions embrace human rights values as their own—a remoralization of the health care system. Human rights education, in other words, seeks to change health care practice by changing what people value, what they feel, what they believe, and it seeks to do so by shifting the frame of reference through which people see themselves and their world, leading them to ask new questions about their own actions and the actions of others. This is the *reflexive* learning of human rights education, where the knowledge and understanding gained is of oneself and one’s context. This objective of human rights education explicitly recognizes that especially in the professions, formal education is a core part of the socialization process that creates normative ways of thinking and acting-the social norms of patient care.

As a cultural project, human rights education focuses on exposing the stereotypes, prejudices, and other harmful social and cultural norms used to rationalize mistreatment of marginalized or vulnerable groups in patient care [1]. As observed by the U.N. Special Rapporteur on Torture, health care practices violent or abusive in their very nature are routinely rationalized on grounds of administrative efficiency, behavior modification, or medical need, which breeds a culture of impunity in health care settings [2, 88]. People who use illicit drugs are denied pain treatment on the basis that it fuels addiction. The competent decisions of people with psycho-social disabilities are disregarded in their supposed best interests. Women living with HIV are sterilized against their will on the belief that they should not bear or parent children. Identified sex workers are subject to mandatory HIV testing and confidentiality breaches for public health protection. These rationalizations reflect a complex amalgam of routinized practice, personal belief, and social norms.

To effect cultural change, human rights education thus seeks to engage with the dominant institutional culture that not only sustains these practices but also leads health providers to think them unproblematic. One aspect of institutional health professional culture that human rights education can tackle is that of strict scientific objectivity, which may bring with it an emotional detachment from everyday life, cutting providers off from valuable sources of information, insight, and understanding about their patients and the conditions of their lives [89]. Human rights education, as a cultural project, is partly an effort to keep health care ‘human.’ The intention is not to devalue biomedical paradigms in health professional education but to recognize and remedy their harmful effects in patient care [76]. A global initiative to integrate reproductive rights into medical education, for example, recommends integrating human rights training with the learning of clinical skills, such as the right to respect for dignity with the learning of breast and pelvic examination techniques that minimize pain and discomfort [50]. A role-play exercise in a third year medical school course in Ankara, Turkey asked students to take on the perspective of patients, to engage with how patients think and feel in a clinical setting [40].

The philosophy and methods of experiential education—that is, the infusion of direct experience into the learning environment—is also a way to encourage patient-provider engagement rather than detachment [90]. Bringing the lived experience of marginalized communities into human rights education, through guest engagements and site visits, can disrupt false but persistent beliefs and norms latent in scientific objective claims of medical or public health need [91]. Where people speak on their own behalf, and give firsthand account of their lives, the stereotypes and prejudices that underlie human rights violations are more difficult to sustain [1, 92]. Health professional education that does not teach ‘about’ people but ‘with’ people reflects the principle of participation, which is core to health and human rights doctrine [93]. In an initiative led by Ipas to promote reproductive rights training in medical and nursing schools in Central America, for example, new physicians credited having more respectful and empathetic attitudes toward their patients to a better understanding of the social determinants underlying the health needs of their patients [59]. A public health course focused on underserved communities on the USA-Mexico border similarly emphasized the connection between students’ growing respect for patients and their learning about the community assets, cultural richness, and historic resilience that families and individuals bring to health encounters [60]. Supplementing direct experience with a more formalized study of social history, drawing on disciplines such as anthropology, sociology, and political history, allows health professionals to further contextualize the conditions of peoples’ lives within larger historical frames of reference [57]. By acknowledging members of marginalized groups as credible and trusted sources of information, by valuing their insights, opinions, and experiences, human rights education not only challenges authoritarian forms of knowledge in professional education but can also fundamentally shift the power dynamic between provider and patient in health care settings. Such experiential human rights education, above all, seeks to foster empathy in an effort to change that moment when a doctor or nurse faces a patient in the examination room and struggles to listen, to understand, and to respect them [94].

International human rights law recognizes the need for cultural change within the health professions to secure equality rights in health care, especially for marginalized and vulnerable groups, and specifically names education as a measure to achieve it. The *U.N. Convention on the Rights of Persons with Disabilities* calls for education measures to ensure “that health professions provide care of the same quality to persons with disabilities … including on the basis of free and informed consent” [95]. The U.N. Convention on the Elimination of all forms of Discrimination against Women similarly requires “that the training curricula of health workers include comprehensive, mandatory, gender-sensitive courses on women’s health and human rights, in particular gender-based violence” [96].

## Human rights education as empowering advocacy

Human rights education as cultural change recognizes the therapeutic value in bearing witness to peoples’ lives, but there is also a social justice agenda in this act [97]. By bearing witness and publicly testifying to human rights violations, health professionals become advocates *for* human rights. In its last and most radical objective, human rights education seeks to build the capacity and skills of health professionals to use their knowledge of how social structures impact on health, knowledge gained through patient care, to advocate for social change [28, 49, 51].

Under this objective, human rights education follows the 19th century tradition of social medicine, famously captured in Virchow’s characterization of the *physician as the natural advocate for the poor* [98]. Through the care provided to patients in community clinics, emergency wards, and operating theaters, health professionals bear direct witness to how social discrimination, abject deprivation, and structural violence shape the health and lives of their patients. In the IPAS initiative on reproductive health and rights education in Central America, graduate physicians expressed their frustration in contraceptive counseling where they were powerless to address gendered social and economic barriers that limited women’s free exercise of the right to decide the number and spacing of their children [59]. Human rights initiatives therefore incorporate human rights advocacy skills within health professional training, especially clinical training, to provide health professionals with the tools to act on a larger scale. Of particular note is human rights education in asylum clinics across the USA, many partnered with the advocacy organization *Physicians for Human Rights*, to provide medical evaluations and affidavits for asylum seekers [41, 42, 56, 99]. In these initiatives, students train and participate in the medico-legal process of asylum seeking, learning how to document health status and needs for the purposes of legal affidavits. The goals of this work are to improve the quality of life for victims of torture and persecution by supporting their efforts to obtain legal asylum, but also, by connecting them with needed health care and other social services. Through clinical practice, moreover, the students and physicians with whom they work learn about and in turn can raise social awareness about the health care and other needs of a deeply vulnerable social group. The asylum clinic example shows that human rights advocacy need not always take a confrontational stance against the state. Health professionals may be equally powerful advocates by collaborating with the state to develop or implement human rights-based programs and policies, such as asylum. What is sometimes referred to as a duty or responsibility of health professionals to act individually and collectively for human rights not only stems from their specialized knowledge but also from their social standing which makes their public statements more likely to be heard and more difficult to ignore [39, 61]. The U.N. Special Rapporteur on the Right to Health has thus endorsed various forms of “practical instruction in how to implement a human rights approach” including “how to identify violations, empower patients or colleagues to defend their human rights, and promote [state] accountability” (para 15) [22].

This is human rights education at its most radical, but also its most contested. Human rights education as empowering advocacy challenges a tradition of professional education as not the engine of social reform, but a potent force in preserving the status quo [45]. Human rights initiatives in health professional education therefore sometimes require and are supported by international donors and developed in partnership with civil society [17, 67]. Even today, human rights education in many contexts retains an outsider status in the health professions, cast as political, confrontational, even unnecessary or inappropriate to professional education as well as professional practice and identity [28, 100]. A study on the teaching of human rights in nursing courses in the UK, for example, noted that prejudice against human rights educators who were also engaged in human rights campaigning or organizing was based on the questionable assumption that their activism undermined the objectivity required in teaching, leading to charges of political bias or even indoctrination [32].

Human rights educators use different strategies to counter professional opposition to their work. Role modeling, for example, can be used to show that human rights advocacy is not a derivation from, but part of a long and celebrated tradition in health professionalism: Black nurses in South Africa who went on strike in 1949 to protest inequalities at the Sulenkama Mission Hospital [55], psychiatrists in Ukraine who spoke out against state psychiatric practices only to be hospitalized themselves as dissidents [74]; the UK Royal College of General Practitioners which issued a policy statement on the right of failed asylum seekers to primary health care [48]. These are all historical examples that can provide role models for students and ideals from which to build a professional identity [101].

Human rights initiatives in health professional education may also seek to support if not embolden faculty members to develop and teach human rights in formal university curriculum recognizing their outsider status. Fostering a *community of practice* is a tool used for this task in human rights education generally, including in health professional education [100]. The human rights program at the University of Cape Town, for example, provides training and in-kind support for health professional educators to develop human rights courses across South Africa and in other African countries [46]. The training includes a specific theme on institutional reform, where alumni share experiences and strategies of seeking institutional support, even protection, for human rights education. OSF supported the development of a similar online community of practice for Eastern Europe and Central Asia, now housed with the Association of Schools of Public Health of the European Region [47, 102].

These communities offer not only an opportunity to develop and share best practices but also a safe and supportive space to build professional alliances and an alternative institutional culture. Yet the need for these alternative communities raises questions about the institutionalization of human rights education in institutions of higher education: does the professionalization of human rights education inevitably lead to its deradicalization? If human rights education is a tool for social justice—to limit state power, to protect against state power, to seize state power—why would institutions of the state foster it, and more importantly, what happens when they do? In many national contexts, government ministries retain tight control over health professional curriculum through the funding, accreditation, and licensing of both public and private institutions. Even more concerning, some state universities discourage faculty and students from expressing their views publicly or participating in public debate, prohibiting political activities [38]. These restrictions make academic freedom, and associated human rights of freedom of expression and association, critical to achieving the radical objectives of human rights education.

Nonetheless, reforms in systems worldwide have opened higher education to new influences. Regulatory measures, like the *Bologna Guidelines* to secure European standards in higher education, have brought opportunity for curricular reform in the region. An elective course on *Discrimination, Health and Globalization*, for example, was introduced at the Faculty of Medicine, University of Geneva, when the faculty adopted these guidelines [44]. Moreover, changing demographics in universities where students begin to more fully reflect a spectrum of social experience can open health professional education to new influences and orientations [60]. Ultimately, one cannot deny nor predict the ways the “university may become … the locus of intellectual resistance and critique, the breeding ground for a human rights and political opposition” [103].

## Conclusions

Human rights education in the health professions carries an ambitious set of objectives: to teach about human rights, to teach through human rights, and to teach for human rights. It entails acquiring knowledge and skills, socializing around values, and producing meaningful change. Human rights education is envisioned as a site of knowledge, change, and justice. This is why human right education is ‘transformative’ in its vision and practice [8]. There is something radical at its roots—human rights education is an effort to realize human rights not in singular, heroic moments, but in systemic reform of the most basic and fundamental institutions of society. These institutions include the health care system, and the guardians of that system, the health professions, and there is no better place to begin their transformation than in the world of patient care.
